# Correction: Expectancy-based rhythmic entrainment as continuous Bayesian inference

**DOI:** 10.1371/journal.pcbi.1009692

**Published:** 2021-12-13

**Authors:** Jonathan Cannon

There are misleading errors in the mathematics deriving an algorithm in S2 Text. The resulting algorithm is unchanged. Please see the correct S2 Text below.

## Supporting information

S2 Text Derivation of filter equations. Mathematical process for deriving the PIPPET and PATIPPET filters from existing point process filtering equations.
(PDF)
