# Supplementary material for: Expectancy-based rhythmic entrainment as continuous Bayesian inference
Source: PLoS Comput Biol. 2021 Jun 9;17(6):e1009025. doi: 10.1371/journal.pcbi.1009025 (PMC8216548; doi:10.1371/journal.pcbi.1009025)
Supplement: S1 Text — Equations describing the filter used to infer both phase and tempo based on point process event timing and a set of event timing expectations over phase. (PDF) [file pcbi.1009025.s001.pdf]

S1 Text: The PATIPPET filter

We let $\boldsymbol{\mu} = \begin{pmatrix} \bar{\phi} \\ \bar{\theta} \end{pmatrix}$ denote the posterior mean and $\mathbf{V} = \begin{pmatrix} V^{11} & V^{12} \\ V^{21} & V^{22} \end{pmatrix}$ denote the posterior covariance. The expressions for the evolution of the PATIPPET filter, which we derive in the following section, are:

$$\begin{cases} d\boldsymbol{\mu} = & \begin{pmatrix} \bar{\theta} \\ 0 \end{pmatrix} dt + (\hat{\boldsymbol{\mu}} - \boldsymbol{\mu}_t) \cdot (dN_t - \Lambda dt) \\ d\mathbf{V} = & \begin{pmatrix} 2V^{12} + \sigma^2 & V^{22} \\ V^{22} & \sigma_\theta^2 \end{pmatrix} dt + \left(\hat{\mathbf{V}} - \mathbf{V}_t\right) \cdot (dN_t - \Lambda dt) \end{cases} \tag{9}$$

where we define

$$\begin{cases} \Lambda := & \sum_{i=0,1,\cdots} \Lambda_i \hat{\theta}_i \\ \hat{\boldsymbol{\mu}} = & \frac{1}{\Lambda} \sum_{i=0,1,\cdots} \Lambda_i \begin{pmatrix} K_i^{12} + \hat{\phi}_i \hat{\theta}_i \\ K_i^{22} + \hat{\theta}_i^2 \end{pmatrix} \\ \hat{\mathbf{V}} := & \frac{1}{\Lambda} \sum_{i=0,1,\cdots} \Lambda_i \Big(\hat{\theta}_i \mathbf{K}_i + \hat{\theta}_i (\hat{\boldsymbol{\mu}}_i - \boldsymbol{\mu}_{t+})(\hat{\boldsymbol{\mu}}_i - \boldsymbol{\mu}_{t+})^T \\ & + (\hat{\boldsymbol{\mu}}_i - \boldsymbol{\mu}_{t+}) \begin{pmatrix} K_i^{21} & K_i^{22} \end{pmatrix} + \begin{pmatrix} K_i^{12} \\ K_i^{22} \end{pmatrix} (\hat{\boldsymbol{\mu}}_i - \boldsymbol{\mu}_{t+})^T \Big) \end{cases} \tag{10}$$

and where

$\mathbf{K}_0 := \mathbf{V}$, $\mathbf{K}_i := \left(\mathbf{P}_i + \mathbf{V}^{-1}\right)^{-1}$ for $i > 0$.

K_i^{kl} denotes the entries in $\mathbf{K}_i$.

$\Lambda_0 := \lambda_0$, $\Lambda_i := \lambda_i \varphi(\phi_i | \bar{\phi}, v_i^{-1} + (V^{11})^{-1})$ for $i > 0$.

$\hat{\boldsymbol{\mu}}_i = \begin{pmatrix} \hat{\phi}_i \\ \hat{\theta}_i \end{pmatrix} := \mathbf{K}_i \left(\begin{pmatrix} v_i^{-1} \phi_i \\ 0 \end{pmatrix} + \mathbf{V}^{-1} \boldsymbol{\mu} \right)$ for $i > 0$, and $\hat{\boldsymbol{\mu}}_0 := \boldsymbol{\mu}$.

$\mathbf{P}_i := \begin{pmatrix} v_i^{-1} & 0 \\ 0 & 0 \end{pmatrix}$
