# Supplementary material for: Expectancy-based rhythmic entrainment as continuous Bayesian inference
Source: PLoS Comput Biol. 2021 Jun 9;17(6):e1009025. doi: 10.1371/journal.pcbi.1009025 (PMC8216548; doi:10.1371/journal.pcbi.1009025)
Supplement: S3 Text — All parameters used in simulation to create the figures above. (PDF) [file pcbi.1009025.s003.pdf]

S3 Text: Simulation parameters.

All code used to create figures in this manuscript is available at https://github.com/joncannon/PIPPET.

PIPPET simulations were conducted by numerical simulation of (3) with $dt = .001$ and initialized with $\phi_0 = 0$ and $V_0 = .0002$. Parameters for the simulations shown in each figure are listed below, with t_n used to denote simulated event times (in units of seconds).

Fig 2: $\phi_1 = .5$, $v_1 = .0005$, $\lambda_1 = 1$, $\mu_t = .43$, $V_t = .001$, $\lambda_0 = 0$ or $.5$, except as otherwise specified.

Fig 3A:

$$\begin{aligned} \{t_n\} =& \{0, .150, .5, .75, .9, 1.25\} \\ \{\phi_i\} =& \{0, .15, .25, .4, .5, .65, .75, .9, 1, 1.15, 1.25, 1.4\} \\ \{v_i\} =& \{.0001, .0005, .0001, .0005, .0001, .0005, .0001, .0005, .0001, .0005\} \\ \{\lambda_i\} =& \{.05, .01, .05, .01, .05, .01, .05, .01, .05, .01\} \\ \lambda_0 =& .01 \\ \sigma =& .05 \end{aligned}$$

Fig 3B: Same as Fig 3A, but with $t_3 = .45$ (50 ms negative event time shift).

Fig 3C: Same as Fig 3A, but with $\{t_n\} = \{0, .15, .5, .7, .85, 1.2\}$ (50 ms negative phase shift).

Fig 4A: Same as Fig 3, but with $\{t_n\} = \{0, .150, .65, .9, 1.15, 1.25\}$.

Fig 4B: Same as Fig 4A, but with $\sigma = .3$.

Fig 4C: Same as Fig 4A, but with additional tap times and tap feedback expectations:

$$\begin{aligned} \{t_n^{tap}\} =& \{\phi_i^{tap}\} = \{0, .5, 1\} \\ v_i^{tap} =& .0005 \\ \lambda_i^{tap} =& .05 \\ \lambda_0^{tap} =& .01 \end{aligned}$$

PATIPPET simulations were conducted by numerical simulation of (4) with $dt = .001$. Parameters for the simulations shown in each figure are listed below.

Fig 5:

$$
\begin{aligned}
t_n =& \frac{n}{1.2Hz} \\
\phi_i =& i \\
v_i =& .005 \\
\lambda_i =& .02 \\
\lambda_0 =& .0001 \\
\sigma =& .05 \\
\sigma_\theta =& .05 \\
\boldsymbol{\mu}_0 =& \begin{pmatrix} 0 \\ 1 \end{pmatrix} \\
\mathbf{V}_0 =& \begin{pmatrix} .001 & 0 \\ 0 & .04 \end{pmatrix}
\end{aligned}
$$

Fig 6: In four simulations, we set the inter-onset interval Δ to $.4s$, $0,7s$, $1.0s$, and $1.3s$. In each simulation, we set the perturbation δ to $\frac{\Delta}{25}$.

$$
\begin{aligned}
\{t_n\} =& \{\Delta, 2\Delta, 3\Delta, 4\Delta + \delta\} \\
\phi_i =& i \\
v_i =& .0002 \\
\lambda_i =& .02 \\
\lambda_0 =& 10^{-5} \\
\sigma =& .01 \\
\sigma_\theta =& .01 \\
\boldsymbol{\mu}_0 =& \begin{pmatrix} 0 \\ 1 \end{pmatrix} \\
\mathbf{V}_0 =& \begin{pmatrix} 10^{-4} & 0 \\ 0 & 10^{-4} \end{pmatrix}
\end{aligned}
$$

Fig 7A:

$$
\begin{aligned}
\phi_i &= .25i \\
v_i &= .0001 \\
\lambda_i &= 1 \\
\lambda_0 &= .0001 \\
\sigma &= .015 \\
\sigma_\theta &= .2 \\
\boldsymbol{\mu}_0 &= \begin{pmatrix} 0 \\ 1 \end{pmatrix} \\
\mathbf{V}_0 &= \begin{pmatrix} .0001 & 0 \\ 0 & .005 \end{pmatrix}
\end{aligned}
$$

Left: $\{t_n\} = \{.25, .5, .75, 1\}$. Right: $\{t_n\} = \{1\}$.

Fig 7B: Same as Fig 7A, but with $\lambda_i = 0$ and $\lambda_0 = 4$.
