# Supplementary material for: Expectancy-based rhythmic entrainment as continuous Bayesian inference
Source: PLoS Comput Biol. 2021 Jun 9;17(6):e1009025. doi: 10.1371/journal.pcbi.1009025 (PMC8216548; doi:10.1371/journal.pcbi.1009025)
Supplement: Attachment — Submitted filename: CannonJ_PlosCB_2020_rev1.pdf [file pcbi.1009025.s004.pdf]

The paper proposes a Bayesian approach to entrainment to complex rhythmic structures. I find this basic framework interesting and may lead to many new developments in temporal inference and rhythmic tracking. The paper is clearly written, and the discussion is extensive. Below are a few points yet to be clarified.

1. The use of the term "entrainment" has created some difficulty for me to process this paper. Perhaps I am clinging to the traditional notion, i.e., the phase and frequency locking between oscillators (the narrow sense in ref.3) – the title gives me the impression that this is the notion adopted in the paper. However, as I read on, this is largely about temporal inference as the "I" in PIPPET and PATIPPET suggests. In some contexts, "entrainment" seems to be used interchangeably with temporal inference, such as in P4-L92 "the problem of entrainment." I hope the author could help readers such as myself by either clarifying early on that here entrainment is used interchangeably with temporal inference, or alternatively, clearly separating the two notions and stating the proposed relation between them. I prefer the latter. More often, "entrainment" is used to refer to the dynamic phenomenon of phase and frequency locking commonly observed in coupled oscillators independent of the specific nature of the coupling functions – thus independent of whether the coupling is of an inferential nature. For example, it would be a bit weird to insist that two pendulum clocks are *inferring* each other's' phase and frequency. Once "entrainment" is defined separately from inference, one can debate whether entrainment is a mechanism for time tracking (objected in ref.3), or in the present context, that the apparent entrainment is an unintended consequence of Bayesian inference based on a periodic likelihood function (how I interpret what author meant).
2. This is somewhat alluded to in Section 4.2, but I wonder whether the author could explicitly discuss whether entrainment to simple v.s. complex rhythmic structure may dependent on separate mechanisms – a more primitive, non-inferential mechanism for the former and an inferential mechanism for the latter (c.f. Comment 1 above). The failure in one may not imply the failure in another (e.g., https://doi.org/10.1016/j.neuropsychologia.2011.02.002). If the inferential mechanism is deemed universal, then the PIPPET and PATIPPET should be benchmarked against more elementary features of entrainment, such as frequency locking (e.g., Peper, Beek, van Wieringen 1995) and out-of-phase to in-phase transition (e.g., Haken, Kelso, & Bunz, 1985).
3. P29-Section 4.3: in PIPPET, the phase ϕ is defined on the real line rather than as a circular variable. Please discussed how the linear phase in PIPPET might be represented in brain oscillations, which I assume, by their circular phases.
4. P16-Fig3A: I notice that the uncertainty at the end is greater than that at the beginning. How does PIPPET maintain bounded uncertainty if the two bars were repeated indefinitely? It would be counterintuitive if the repetition of the same material leads to worse phase estimation. Please discuss.
5. Section 3.7: since we are in the Results, one would expect to see an example solution to the tracking of multi-stream rhythms. It seems that only the template is given. Please show an example of the dynamics of the posterior like those in Figure3-5.

Minor points:

6. In P4-L87, the author defines “stochastic” rhythm as “rhythms in which some expected events may [be] omitted.” This may not be how most people interpret “stochasticity.” The term in the title actually misled me to look for results based on notes stochastically generated by a periodic likelihood function. I suggest simply using “complex rhythmic patterns” or “complex event sequences” to prevent misunderstanding.
7. P7-eq2: I think it should be stated early on, e.g., in this subsection, that the likelihood function is given/known to the observer.
8. P7-L173: could you write the posterior distribution explicitly in the form of the conditional probability? It would be easier to read.
9. P8-Fig1: if $\lambda(\phi)$ is the likelihood function, shouldn’t it be less or equal to 1 for all ϕ?
10. P11-L227: you meant “θ” (not T) is the rate of phase advancement.
11. P11-L231: you meant $\lambda(\phi)$?
12. P13-L274: Figure 2B?
13. P15-Fig3: Please move the explanation of the color code, lines, shaded areas from Figure 5 to here.
