# Supplementary material for: Expectancy-based rhythmic entrainment as continuous Bayesian inference
Source: PLoS Comput Biol. 2021 Jun 9;17(6):e1009025. doi: 10.1371/journal.pcbi.1009025 (PMC8216548; doi:10.1371/journal.pcbi.1009025)
Supplement: Attachment — Submitted filename: PIPPET reviewer responses.pdf [file pcbi.1009025.s005.pdf]

Dear editors and reviewers,

This manuscript and I have both benefited hugely from the biological and experimental thinking you have pushed us to take on.

To provide clear biological insights and criteria for falsification, I found it necessary to reframe the model, highlighting the qualitative aspects that made it novel (uncertainty tracking and the influence of unfulfilled expectations) in addition to its normative grounding. This required significant revision throughout the manuscript, to the point that it no longer made sense to simply highlight the changes. The mathematical and computational results are largely unchanged, with a few key exceptions:

- **For Figures 3 and 4, subpanels were introduced to highlight the key role of uncertainty.**
- **The tempo-tracking framework was revised by introducing a direct dependency of event rate on tempo into the generative model. This is justified in the text, and leads to a new result in Figure 7: even a general expectancy of events without temporal specificity can induce filled duration illusions.**

I added section 4.6 and Figure 8 to align the normative model with neurophysiology. If this level of biological connection is not sufficient to suit the manuscript to PLOS Comp Bio, I will have to submit it elsewhere. However, I believe that there is now real biological insight to be gleaned from this work.

Additional substantial but aesthetic changes: Figure 1 is now an extensive illustration of the mathematical components of the model, and in Figure 2 I have replaced the phase transition and response curves with thorough explorations of the effect of parameters and initial phase distribution on posterior distribution after an event. I also changed some of the notation throughout for better mathematical legibility.

Specific responses to reviewers are included in boldface below. Whether or not the paper is published with PLOS, your feedback will have been invaluable. Thank you,

Jonathan Cannon

P.S. As instructed, I am noting here that I am including the Methods section *before* the Results section because I believe that a discussion of the models is necessary to make the results comprehensible.

Dear Dr. Cannon,

Thank you very much for submitting your manuscript "PIPPET: A Bayesian framework for generalized entrainment to stochastic rhythms" for consideration at PLOS Computational Biology.

As with all papers reviewed by the journal, your manuscript was reviewed by members of the editorial board and by several independent reviewers. In light of the reviews (below this email), we would like to invite the resubmission of a significantly-revised version that takes into account the reviewers' comments.

The reviewers have raised some very important issues that must be thoroughly addressed to render this paper suitable for PLoS Computational Biology. If they are beyond the scope of this paper, then this manuscript may be more suitable for a journal oriented to more purely theoretical work. But if they can be addressed, then we will be happy to consider the revised MS for PLoS CB. Specifically, the reviewers appropriately ask

- that a neurally mechanistic implementation for the model should be presented

Added Section 4.6 and Figure 8

- that the biological insights gained from the work should be clearly stated

This was addressed by discussing possible biological mechanisms and focusing on the representation of uncertainty in the brain.

- that additional parametric analysis of the model and how it functions should be provided

This is now provided in Figure 2

- that experiments that could be used to falsify the model should be proposed.

These are now discussed in section 4.3

Of course, the reviewers' additional comments should also be addressed in revision.

We cannot make any decision about publication until we have seen the revised manuscript and your response to the reviewers' comments. Your revised manuscript is also likely to be sent to reviewers for further evaluation.

When you are ready to resubmit, please upload the following:

[1] A letter containing a detailed list of your responses to the review comments and a description of the changes you have made in the manuscript. Please note while forming your response, if your article is accepted, you may have the opportunity to make the peer review history publicly

available. The record will include editor decision letters (with reviews) and your responses to reviewer comments. If eligible, we will contact you to opt in or out.

[2] Two versions of the revised manuscript: one with either highlights or tracked changes denoting where the text has been changed; the other a clean version (uploaded as the manuscript file).

Important additional instructions are given below your reviewer comments.

Please prepare and submit your revised manuscript within 60 days. If you anticipate any delay, please let us know the expected resubmission date by replying to this email. Please note that revised manuscripts received after the 60-day due date may require evaluation and peer review similar to newly submitted manuscripts.

Thank you again for your submission. We hope that our editorial process has been constructive so far, and we welcome your feedback at any time. Please don't hesitate to contact us if you have any questions or comments.

Sincerely,

Jonathan Rubin

Associate Editor

PLOS Computational Biology

Samuel Gershman

Deputy Editor

PLOS Computational Biology

****

Reviewer's Responses to Questions

Comments to the Authors:

Reviewer #1: In the current work the author introduces a Bayesian framework describing the human ability to entrain to an auditory stimulus. The work is clearly written, the model is well formulated and the author shows that the proposed framework qualitatively reproduces a set of empirical observations. However, the current study does not reach the required biological or methodological insight to be published in PLOS Comp. Biol. While it is a well sound work it belongs to a more mathematically (or modelling) oriented Journal.

While the proposed framework is compatible with some observations it is not clear how it can be mechanistically implemented at brain level neither can the author propose a feasible experiment capable of falsifying the model. Specifically, some abstractions of the model reducing its biological relevance are: the parameters are arbitrary adjusted with no clear biophysical interpretation, the predictions are not quantitatively compared with experimental data, there is no commitment for a neural mechanism, neither for a brain area, responsible for the Bayesian estimation, the same framework informs production and perception neglecting the interaction between the systems. Given that the framework is disconnected from the underlying biological process it is not clear for this Reviewer which are the new insights of this study on how the brain mechanistically works.

It is possible that this work would have been well suited to a less biologically-oriented journal. However, your points encouraged me to more deeply explore the experimental testability and biological implementation of these ideas. I now discuss both points thoroughly in the DIscussion section. If these discussions are not sufficient to suit this work to PLOS Comp Bio, so be it -- I will submit elsewhere, and will appreciate the ways in which your feedback encouraged me to deepen my thinking. But I now believe that these ideas are behaviorally testable [section 4.3] and point toward specific implementation in the brain [section 4.6 and Figure 8].

Author states in the Introduction that oscillator like theories describe predictions about the "what" rather than the "when". There are, however, examples in the literature where oscillator like models are used to make temporal predictions (see for example Assaneo, Rimmele et al. 2020 Nat. Hum. Beh.)

The passage you refer to was intended to refer to predictive coding models, not oscillator-like theories, which do indeed make temporal predictions. The passage read:

> *"Predictive processing would be a natural modeling framework for understanding rhythmic expectation and entrainment as inference \cite{Vuust2014, Vuust2018, Proksch2020} except for one key limitation: existing predictive coding models that operate in continuous time are structured to perform inference based on continuous observation, characterizing prediction errors in terms of deviation between a true level of input and a mean expected level of input \cite{Friston2010, Buckley2017}. They describe predictions about ``what'' rather than ``when,'' and are therefore ill-suited to characterizing moment-by-moment errors in timing prediction, which arrive sporadically, separated by intervals largely devoid of informative prediction error."*

Hopefully this wording is clear.

Reviewer #2:

The paper proposes a Bayesian approach to entrainment to complex rhythmic structures. I find this basic framework interesting and may lead to many new developments in temporal inference and rhythmic tracking. The paper is clearly written, and the discussion is extensive. Below are a few points yet to be clarified.

1. The use of the term “entrainment” has created some difficulty for me to process this paper. Perhaps I am clinging to the traditional notion, i.e., the phase and frequency locking between oscillators (the narrow sense in ref.3) – the title gives me the impression that this is the notion adopted in the paper. However, as I read on, this is largely about temporal inference as the “I” in PIPPET and PATIPPET suggests. In some contexts, “entrainment” seems to be used interchangeably with temporal inference, such as in P4-L92 “the problem of entrainment.” I hope the author could help readers such as myself by either clarifying early on that here entrainment is used interchangeably with temporal inference, or alternatively, clearly separating the two notions and stating the proposed relation between them. I prefer the latter. More often, “entrainment” is used to refer to the dynamic phenomenon of phase and frequency locking commonly observed in coupled oscillators independent of the specific nature of the coupling functions – thus independent of whether the coupling is of an inferential nature. For example, it would be a bit weird to insist that two pendulum clocks are inferring each other’s’ phase and frequency. Once “entrainment” is defined separately from inference, one can debate whether entrainment is a mechanism for time tracking (objected in ref.3), or in the present context, that the apparent entrainment is an unintended consequence of Bayesian inference based on a periodic likelihood function (how I interpret what author meant).

Thank you for pointing out this critical distinction! You are right that what I am presenting here is a very specific type of entrainment -- it is entrainment optimized to serve a very specific purpose. I have coined and broadly introduced the term “expectation-” or “expectancy-based entrainment” (including in the title) to differentiate this from general entrainment.

2. This is somewhat alluded to in Section 4.2, but I wonder whether the author could explicitly discuss whether entrainment to simple v.s. complex rhythmic structure may dependent on separate mechanisms – a more primitive, non-inferential mechanism for the former and an inferential mechanism for the latter (c.f. Comment 1 above). The failure in one may not imply the failure in another (e.g., https://doi.org/10.1016/j.neuropsychologia.2011.02.002). If the inferential mechanism is deemed universal, then the PIPPET and PATIPPET should be benchmarked against more elementary features of entrainment, such as frequency locking (e.g., Peper, Beek, van Wieringen 1995) and out-of-phase to in-phase transition (e.g., Haken, Kelso, & Bunz, 1985).

Excellent point. In section 4.5.1, I now discuss how the model may not be a good description of the rapid, automatic adjustments to motor entrainment that may be implemented by lower motor structures.

3. P29-Section 4.3: in PIPPET, the phase ϕ is defined on the real line rather than as a circular variable. Please discussed how the linear phase in PIPPET might be represented in brain oscillations, which I assume, by their circular phases.

Putting the phase on the line was a choice made for flexibility and tractability, and for simple cyclic patterns I agree that we should expect to see phase represented circularly. However, with help from the basal ganglia over longer time scales, cylinders and spirals may be possible (Russo et al 2020)! I now address this point on line 611.

4. P16-Fig3A: I notice that the uncertainty at the end is greater than that at the beginning. How does PIPPET maintain bounded uncertainty if the two bars were repeated indefinitely? It would be counterintuitive if the repetition of the same material leads to worse phase estimation. Please discuss.

Perhaps the image was misleading -- events aligned with precise expectations can easily keep uncertainty bounded. Throughout the revised text, including in the new Fig 3 caption, I try to be more explicit about keeping uncertainty bounded.

5. Section 3.7: since we are in the Results, one would expect to see an example solution to the tracking of multi-stream rhythms. It seems that only the template is given. Please show an example of the dynamics of the posterior like those in Figure3-5.

Good point. Rather than creating a contrived example of multi-voiced rhythms, I cut this section, and applied mPIPPET to make a larger point about uncertainty management by adding a tapping-along example to FIgure 4.

Minor points:

6. In P4-L87, the author defines “stochastic” rhythm as “rhythms in which some expected events may [be] omitted.” This may not be how most people interpret “stochasticity.” The term in the title actually misled me to look for results based on notes stochastically generated by a periodic likelihood function. I suggest simply using “complex rhythmic patterns” or “complex event sequences” to prevent misunderstanding.

Good point. I have changed this throughout to “complex rhythms.”

7. P7-eq2: I think it should be stated early on, e.g., in this subsection, that the likelihood function is given/known to the observer.
Added this on line 132.

8. P7-L173: could you write the posterior distribution explicitly in the form of the conditional probability? It would be easier to read.
Added this on line 155.

9. P8-Fig1: if $\lambda(\phi)$ is the likelihood function, shouldn't it be less or equal to 1 for all ϕ?
Good catch. lambda(phi)dt is a likelihood function, making lambda(phi) a rescaled likelihood function. I now specify that on line 148.

10. P11-L227: you meant "θ" (not T) is the rate of phase advancement.
Typo fixed

11. P11-L231: you meant $\lambda(\phi)$?
Typo was removed in revision process

12. P13-L274: Figure 2B?
Typo fixed

13. P15-Fig3: Please move the explanation of the color code, lines, shaded areas from Figure 5 to here.
Corrected

Reviewer #3: This study proposes three models, PIPPET, PATIPPET and m-PIPPET using random diffusion variables to continuously estimate the underlying phase and tempo of beats with simple and complex metrics based on precise event times and their correspondence with timing expectations. These models estimate the posterior probability of phase and tempo using a likelihood distribution that represents a temporal expectation template and a prior function that represents the phase/tempo before to estimation. For PIPPET, the likelihood distribution is the sum of a linear combination of Gaussian functions centered on training phases, and a bias term. This function allows to consider different types of expected rhythmic events, such as isochronous or complex beats by adjusting the coefficients of the likelihood function. In addition, the a priori distribution is a Gaussian distribution with mean and variance equal to mean and variance of posterior distribution of the previous phase, this maintains a memory process over time.

Interestingly, the Snyder's theory is used to estimate the mean and variance of the posterior distribution in a continuously manner using the likelihood and the prior. The posterior distribution changes with the occurrence of an event as follows: 1) If an event is very close to a high expected phase, then the variance is reduced, emulating a restart of time processing. 2) if the event occurs in an expected phase neighborhood, the mean distribution is readjusted to the expected phase, emulating a time step readjustment. And 3) if an event occurs far from any expected phase, it has no effect on posterior distribution, this is convenient because in the absence of an event, the phase variable follows the course of time. These three elements reply synchronous or asynchronous rhythms and expected templates and describes very well time distortions.

For the PATIPPET model, the estimate of the expected interval duration is added. A re-adjustment in duration is observed minimizing error and temporal uncertainty. These models reproduce period-dependent phase corrections, illusory contraction of unexpectedly empty intervals, and failure to track excessively syncopated rhythms. In conclusion, this work shows an innovative model for continuously estimating the passage of time and duration of the interval between rhythmic events.

This is an interesting and well written and properly structured manuscript providing a novel set of models to continuously estimate performance on a wide range of timing and entrainment tasks, incorporating active inference on rhythmic timing. My main concerns on the paper are the following. First, there is no parametric analysis of the model variables and their impact on the solution of the posterior probabilities.

This has been added as Figure 2.

In addition, a graphical representation of the intuitions described in page 10 would be very useful.

Excellent point. This has been added as Figure 1, and (I hope) has significantly improved the model's comprehensibility and the clarity of my own thinking about it.

Second, although the diffusion process has been well accepted biologically, with ramping activity of neurons as a plausible neural correlate, it is not clear how the brain can compute the mean and variance of the likelihood function, what is the neural correlate of the prior and how the posterior distribution can be computed from these two independent signals.

Is not clear neither whether these computations depend on changes in discharge rate of a network, on the state dynamics of neural populations, and/or on the changes in power of oscillatory local oscillatory activity (see Balasubramaniam et al., 2020 J Neurosci). The author claims that the proposed models are more physiologically plausible than previous ones; however, this is not evident. In fact, the actual hypothesis is that rhythmic perception and entrainment depends on the close interaction of the audiomotor system, with a predictive signal generated in the motor system and a sensory input in the auditory cortex subject of active inference and error correction. The model integrates prediction, inference and error correction in the same system with no compartmentalization in the processing.

This is a challenging problem, and one that I cannot treat thoroughly in the confines of this manuscript. However, I have added Section 4.6 and Figure 8, which attempt to align the phase inference framework with a possible neurophysiological implementation.

In addition, the author simulates experiments of beat perception and beat entrainment interchangeably with the models, when we know there is clear differences in the neural processing (Merchant et al., 2015 Philos Trans R Soc Lond B Biol Sci).

This is now discussed in 4.5.1. I account for the differences in terms of additional mechanisms employed to rapidly correct movement trajectory, which may not be well-described by what is essentially a perceptual model. However, I think it's clear that our malleable, expectation-based perceptual sense of temporal structure strongly influences motor entrainment, as is evident in situations where time-shifted subdivisions influence tap time.

Furthermore, the time lag of the prior is only -1, what happens when you have different and multiple lags.

I'm not totally sure I understand this question. In section 4.6 I now discuss how any neurophysiological implementation of the phase inference framework would have to dispense with the mathematical convenience of instantaneous phase updates. I do think that it's reasonable to describe phase inference by a Markov-like process, where all information necessary for the next time step is present at this time step.

Finally, the paper does not include a section on how the model parameters can be adjusted to experimental data, which can give a clear intuition on how the models are built.

This is now discussed in section 4.2 (how to understand the "meaning" of model parameters) and 4.4 (with a specific example of how parameters may be redundant for some experiments and how you might disentangle them).

Minor comments.

Please change the color for ∑t- and ∑t+ for Figure 2B.

Figure 2 has been thoroughly revised and color coded.

A new panel in Figure 2 with ∑ and log() as a function of t could be very use full.

Figures 3, 4 and 5 should have a panel ∑ as a function of rhythm and expectation.

Uncertainty is represented by shading in these figures. A key has been added for clarityy.

The legend of figure 3 instead of figure 5 should describe the specifics of the panels content.

Fixed.
