# Supplementary material for: Correction: Expectancy-based rhythmic entrainment as continuous Bayesian inference
Source: PLoS Comput Biol. 2021 Dec 13;17(12):e1009692. doi: 10.1371/journal.pcbi.1009692 (PMC8668110; doi:10.1371/journal.pcbi.1009692)
Supplement: S2 Text — Mathematical process for deriving the PIPPET and PATIPPET filters from existing point process filtering equations. (PDF) [file pcbi.1009692.s001.pdf]

S2 Text: Derivation of filter equations.

Here we derive the PATIPPET filter; the PIPPET filter can be derived similarly or as a special case of PATIPPET.

Snyder [1] provides a partial differential equation describing the evolution of a probability distribution on a continuously stochastically evolving state that drives the emission of point process events. If the evolution of the underlying state is described by a Gauss-Markov diffusion process:

$$d\mathbf{x} = \mathbf{A}\mathbf{x}dt + \mathbf{B}d\mathbf{W}_t \tag{11}$$

and events are generated at rate $\lambda(\mathbf{x})$, then the evolution of the probability distribution $p_t(\mathbf{x})$ is described by

$$dp_t(\mathbf{x}) = \mathcal{L}[p_t(\mathbf{x})]dt + p_t(\mathbf{x})\left(\frac{\lambda(\mathbf{x})}{\Lambda} - 1\right) \cdot (dN_t - \Lambda dt) \tag{12}$$

where $\Lambda := \mathbb{E}[\lambda(\mathbf{x})]$ (with $\mathbb{E}$ denoting expectation under distribution $p_t(\mathbf{x})$), dN_t is the increment in the event count over each dt time step (assumed to be either 1 or 0 with probability 1), and $\mathcal{L}$ is the Kolmogorov forward operator associated with (11):

$$\mathcal{L}[p(\mathbf{x})] = -\sum_i \frac{\partial}{\partial x_i}[\mathbf{A}\mathbf{x}]_i p(\mathbf{x}) + \frac{1}{2}\sum_{i,j}\frac{\partial^2}{\partial x_i \partial x_j}[\mathbf{B}\mathbf{B}^T]_{ij} p(\mathbf{x}) \tag{13}$$

Here we project p onto a Gaussian distribution at each time step by matching mean $\boldsymbol{\mu}$ and covariance $\mathbf{V}$, which is also the projection with minimal KL divergence. We do this by finding the differentials of these moments of p_t and using them to drive the evolution of these two variables:

$$\begin{aligned} d\boldsymbol{\mu}_t =& \boldsymbol{\mu}_{t+} - \boldsymbol{\mu}_t = \int_{\mathbf{x}} \mathbf{x} p_{t+}(\mathbf{x}) d\mathbf{x} - \int_{\mathbf{x}} \mathbf{x} p_t(\mathbf{x}) d\mathbf{x} \\ =& \int_{\mathbf{x}} \mathbf{x} \left(p_{t+}(\mathbf{x}) - p_t(\mathbf{x})\right)(\mathbf{x}) d\mathbf{x} = \int_{\mathbf{x}} \mathbf{x} dp_t(\mathbf{x}) d\mathbf{x} \\ =& \int_{\mathbf{x}} \mathbf{x} \mathcal{L}[p_t(\mathbf{x})] dt d\mathbf{x} + (\hat{\boldsymbol{\mu}} - \boldsymbol{\mu}_t) \cdot (dN_t - \Lambda dt) \end{aligned} \tag{14}$$

where we define $\hat{\boldsymbol{\mu}} := \frac{1}{\Lambda}\mathbb{E}\left[\mathbf{x}\lambda(\mathbf{x})\right]$, and

$$d\mathbf{V}_t = \mathbf{V}_{t+} - \mathbf{V}_t = \int_{\mathbf{x}} [[\mathbf{x} - \boldsymbol{\mu}_{t+}]]^2 p_{t+}(\mathbf{x}) d\mathbf{x} - \int_{\mathbf{x}} [[\mathbf{x} - \boldsymbol{\mu}_t]]^2 p_t(\mathbf{x}) d\mathbf{x}$$

where $[[\mathbf{x}]]^2$ denotes $\mathbf{x}\mathbf{x}^T$.

$$\begin{aligned} d\mathbf{V}_t &= \int_{\mathbf{x}} [[\mathbf{x} - \boldsymbol{\mu}_{t+}]]^2 \left(p_{t+}(\mathbf{x}) - p_t(\mathbf{x})\right) d\mathbf{x} \\ &\quad + \int_{\mathbf{x}} \left([[\mathbf{x} - \boldsymbol{\mu}_{t+}]]^2 - [[\mathbf{x} - \boldsymbol{\mu}_t]]^2\right) p_t(\mathbf{x}) d\mathbf{x} \\ &= \int_{\mathbf{x}} [[\mathbf{x} - \boldsymbol{\mu}_{t+}]]^2 dp_t(\mathbf{x}) - [[\boldsymbol{\mu}_{t+} - \boldsymbol{\mu}_t]]^2 \\ &= \int_{\mathbf{x}} [[\mathbf{x} - \boldsymbol{\mu}_{t+}]]^2 \mathcal{L}[p_t(\mathbf{x}|N_t)] dt d\mathbf{x} + \left(\hat{\mathbf{V}} - \mathbf{V}_t\right) \cdot (dN_t - \Lambda dt) \end{aligned} \tag{15}$$

where we define $\hat{\mathbf{V}} := \frac{1}{\Lambda}\mathbb{E}\left[[[\mathbf{x} - \boldsymbol{\mu}_{t+}]]^2 \lambda(\mathbf{x})\right]$.

Integrating by parts (or following [3]), we can calculate the appropriate integrals of $\mathcal{L}[p_t(\mathbf{x}|N_t)]$, arriving at a general expression for the variational Bayesian filter for point process data:

$$\begin{cases} d\boldsymbol{\mu}_t = & \mathbf{A}\boldsymbol{\mu}_t dt + (\hat{\boldsymbol{\mu}} - \boldsymbol{\mu}_t) \cdot (dN_t - \Lambda dt) \\ d\mathbf{V}_t = & (\mathbf{A}\mathbf{V}_t + \mathbf{V}_t\mathbf{A}^T + \mathbf{B}\mathbf{B}^T)dt + \left(\hat{\mathbf{V}} - \mathbf{V}_t\right) \cdot (dN_t - \Lambda dt) \end{cases} \tag{16}$$

From (4), the PATIPPET generative model is described by the Gauss-Markov diffusion process (11) with

$$\mathbf{x} = \begin{pmatrix} \phi \\ \theta \end{pmatrix} \text{ and } \boldsymbol{\mu} = \begin{pmatrix} \bar{\phi} \\ \bar{\theta} \end{pmatrix}$$

$$\mathbf{V} = \begin{pmatrix} V^{11} & V^{12} \\ V^{21} & V^{22} \end{pmatrix}$$

$$\mathbf{A} := \begin{pmatrix} 0 & 1 \\ 0 & 0 \end{pmatrix} \text{ and } \mathbf{B} := \begin{pmatrix} \sigma & 0 \\ 0 & \sigma_\theta \end{pmatrix}.$$

Plugging into (16), we have

$$\begin{cases} d\boldsymbol{\mu}_t = & \begin{pmatrix} \bar{\theta} \\ 0 \end{pmatrix} dt + (\hat{\boldsymbol{\mu}} - \boldsymbol{\mu}_t) \cdot (dN_t - \Lambda dt) \\ d\mathbf{V} = & \begin{pmatrix} 2V^{12} + \sigma^2 & V^{22} \\ V^{22} & \sigma_\theta^2 \end{pmatrix} dt + \left(\hat{\mathbf{V}} - \mathbf{V}_t\right) \cdot (dN_t - \Lambda dt) \end{cases} \tag{17}$$

We complete the derivation by calculating Λ, $\hat{\boldsymbol{\mu}}$, and $\hat{\mathbf{V}}$. This proceeds by first deriving a simple expression

for $p(\mathbf{x})\lambda(\mathbf{x})$ as a sum of scaled normal distributions.

Let $\|x\|_A^2$ denote $x^T A x$. We will make use of the following result, a generalized form of a well-known result about quadratic forms that allows us to write products of multivariate normal distributions as normal distributions (see [2] for proof and similar application):

$$\|x-a\|_A^2 + \|x-b\|_B^2 = \|a-b\|_{A(A+B)^{-1}B}^2 + \|x-(A+B)^{-1}(Aa+Bb)\|_{A+B}^2 \tag{18}$$

In the PATIPPET generative model, events are generated at rate

$$\lambda(\mathbf{x}) = \theta\left(\lambda_0 + \sum_{i=1,2,\cdots} \frac{\lambda_i}{\sqrt{2\pi v_i}} e^{-\frac{1}{2}\|\mathbf{x}-\mathbf{x}_i\|_{\mathbf{P}_i}^2}\right)$$

$$\mathbf{P}_i = \begin{pmatrix} v_i^{-1} & 0 \\ 0 & 0 \end{pmatrix},\ \mathbf{x}_i = \begin{pmatrix} \phi_i \\ 0 \end{pmatrix}.$$

$p(\mathbf{x})$ is assumed (forced) to be Gaussian, so we can write:

$$p(\mathbf{x}) = \frac{1}{\sqrt{2\pi|\mathbf{V}|}} e^{-\frac{1}{2}\|\mathbf{x}-\boldsymbol{\mu}\|_{\mathbf{V}^{-1}}^2}.$$

We calculate:

$$\begin{aligned} p(\mathbf{x})\lambda(\mathbf{x}) =& \frac{\theta}{\sqrt{2\pi|\mathbf{V}|}} e^{-\frac{1}{2}\|\mathbf{x}-\boldsymbol{\mu}\|_{\mathbf{V}^{-1}}^2}\left(\lambda_0 + \sum_{i=1,2,\cdots} \frac{\lambda_i}{\sqrt{2\pi v_i}} e^{-\frac{1}{2}\|\mathbf{x}-\mathbf{x}_i\|_{\mathbf{P}_i}^2}\right) \\ =& \frac{\lambda_0\theta}{\sqrt{2\pi|\mathbf{V}|}} e^{-\frac{1}{2}\|\mathbf{x}-\boldsymbol{\mu}\|_{\mathbf{V}^{-1}}^2} + \theta \sum_{i=1,2,\cdots} \frac{\lambda_i}{2\pi\sqrt{v_i|\mathbf{V}|}} e^{-\frac{1}{2}\|\mathbf{x}-\mathbf{x}_i\|_{\mathbf{P}_i}^2 - \frac{1}{2}\|\mathbf{x}-\boldsymbol{\mu}\|_{\mathbf{V}^{-1}}^2} \end{aligned}$$

Applying (18),

$$\begin{aligned} p(\mathbf{x})\lambda(\mathbf{x}) =& \frac{\lambda_0\theta}{\sqrt{2\pi|\mathbf{V}|}} e^{-\frac{1}{2}\|\mathbf{x}-\boldsymbol{\mu}\|_{\mathbf{V}^{-1}}^2} \\ &+ \theta \sum_{i=1,2,\cdots} \lambda_i \left(\frac{1}{\sqrt{2\pi(v_i+V^{11})}} e^{-\frac{1}{2}\|\mathbf{x}_i-\boldsymbol{\mu}\|_{\mathbf{P}_i\mathbf{K}_i\mathbf{V}^{-1}}^2}\right)\left(\frac{1}{\sqrt{2\pi\frac{v_i|\mathbf{V}|}{v_i+V^{11}}}} e^{-\frac{1}{2}\|\mathbf{x}-\mathbf{K}_i(\mathbf{P}_i\mathbf{x}_i+\mathbf{V}^{-1}\boldsymbol{\mu})\|_{\mathbf{K}_i^{-1}}^2}\right) \end{aligned} \tag{19}$$

where we define $\mathbf{K}_i := (\mathbf{P}_i + \mathbf{V}^{-1})^{-1}$. It can be quickly verified that the first of the two final terms represents the PDF of a univariate normal distribution on ϕ_i, while the second represents the PDF of a multivariate

normal distribution on $\mathbf{x}$. Thus, we can rewrite (19) as

$$p(\mathbf{x})\lambda(\mathbf{x}) = \lambda_0\theta\varphi(\mathbf{x}|\boldsymbol{\mu},\mathbf{V}) + \theta \sum_{i=1,2,\cdots} \lambda_i\varphi(\phi_i|\bar{\phi}, v_i + V^{11})\varphi(\mathbf{x}|\mathbf{K}_i(\mathbf{P}_i\mathbf{x}_i + \mathbf{V}^{-1}\boldsymbol{\mu}), \mathbf{K}_i) \tag{20}$$

We simplify this expression by defining $\Lambda_i := \lambda_i\varphi(\phi_i|\bar{\phi}, v_i + V^{11})$ for $i > 0$, and setting $\Lambda_0 := \lambda_0$ and $\mathbf{K}_0 = \mathbf{V}$. We define $\hat{\boldsymbol{\mu}}_i := \begin{pmatrix} \hat{\phi}_i \\ \hat{\theta}_i \end{pmatrix} := \mathbf{K}_i(\mathbf{P}_i\mathbf{x}_i + \mathbf{V}^{-1}\boldsymbol{\mu})$ for $i > 0$ and set $\hat{\boldsymbol{\mu}}_0 := \boldsymbol{\mu}$. This lets us write

$$p(\mathbf{x})\lambda(\mathbf{x}) = \sum_{i=0,1,\cdots} \Lambda_i\theta\varphi(\mathbf{x}|\hat{\boldsymbol{\mu}}_i, \mathbf{K}_i) \tag{21}$$

We use this expression and the moments of normal distributions to calculate Λ, $\hat{\boldsymbol{\mu}}$, and $\hat{\mathbf{V}}$:

$$\Lambda := \mathbb{E}_p\left[\lambda(\mathbf{x})\right] = \sum_{i=0,1,\cdots} \Lambda_i \int \theta\varphi(\mathbf{x}|\hat{\boldsymbol{\mu}}_i, \mathbf{K}_i)d\mathbf{x} = \sum_{i=0,1,\cdots} \Lambda_i\hat{\theta}_i \tag{22}$$

$$\hat{\boldsymbol{\mu}} := \frac{1}{\Lambda}\mathbb{E}\left[\mathbf{x}\lambda(\mathbf{x})\right] = \frac{1}{\Lambda}\sum_{i=0,1,\cdots} \Lambda_i \int \begin{pmatrix} \phi\theta \\ \theta^2 \end{pmatrix} \varphi(\mathbf{x}|\hat{\boldsymbol{\mu}}_i, \mathbf{K}_i)d\mathbf{x}$$

This expression picks out non-central second moment terms of each normal distributions in (21), each of which can be written in terms of the covariance matrix and mean of the distribution. Using K_i^{kl} to denote the entries in $\mathbf{K}_i$, we can write

$$\hat{\boldsymbol{\mu}} = \frac{1}{\Lambda}\sum_{i=0,1,\cdots} \Lambda_i \begin{pmatrix} K_i^{12} + \hat{\phi}_i\hat{\theta}_i \\ K_i^{22} + \hat{\theta}_i^2 \end{pmatrix} \tag{23}$$

The third-order expression for $\hat{\mathbf{V}}$ can also be written in terms of covariance matrices and means since the central third moments of normal distributions are zero.

$$\begin{aligned}
\hat{\mathbf{V}} :=& \frac{1}{\Lambda}\mathbb{E}_p\left[[[\mathbf{x}-\boldsymbol{\mu}_{t+}]]^2\lambda(\mathbf{x})\right] \\
=& \frac{1}{\Lambda}\sum_{i=0,1,\cdots}\Lambda_i\int[[\mathbf{x}-\boldsymbol{\mu}_{t+}]]^2\theta\varphi(\mathbf{x}|\hat{\boldsymbol{\mu}}_i,\mathbf{K}_i)d\mathbf{x} \\
=& \sum_{i=0,1,\cdots}\Lambda_i\Big[\hat{\theta}_i\int[[\mathbf{x}-\hat{\boldsymbol{\mu}}_i]]^2\varphi(\mathbf{x}|\hat{\boldsymbol{\mu}}_i,\mathbf{K}_i)d\mathbf{x}\cdots \\
&+\hat{\theta}_i[[\hat{\boldsymbol{\mu}}_i-\boldsymbol{\mu}_{t+}]]^2\cdots \\
&+(\hat{\boldsymbol{\mu}}_i-\boldsymbol{\mu}_{t+})\int(\mathbf{x}-\hat{\boldsymbol{\mu}}_i)^T(\theta-\hat{\theta}_i)\varphi(\mathbf{x}|\hat{\boldsymbol{\mu}}_i,\mathbf{K}_i)d\mathbf{x}\cdots \\
&+\left(\int(\mathbf{x}-\hat{\boldsymbol{\mu}}_i)(\theta-\hat{\theta}_i)\varphi(\mathbf{x}|\hat{\boldsymbol{\mu}}_i,\mathbf{K}_i)d\mathbf{x}\right)(\hat{\boldsymbol{\mu}}_i-\boldsymbol{\mu}_{t+})^T\Big] \\
=& \frac{1}{\Lambda}\sum_{i=0,1,\cdots}\Lambda_i[\hat{\theta}_i\mathbf{K}_i+\hat{\theta}_i[[\hat{\boldsymbol{\mu}}_i-\boldsymbol{\mu}_{t+}]]^2\cdots \\
&+(\hat{\boldsymbol{\mu}}_i-\boldsymbol{\mu}_{t+})\begin{pmatrix}K_i^{21} & K_i^{22}\end{pmatrix}+\begin{pmatrix}K_i^{12}\\ K_i^{22}\end{pmatrix}(\hat{\boldsymbol{\mu}}_i-\boldsymbol{\mu}_{t+})^T]
\end{aligned} \tag{24}$$

Expressions (22), (23), and (24) coupled with (17) constitute the PATIPPET filter.

The PIPPET filter can be derived as a special case of the PATIPPET filter by setting $\sigma_\theta = 0$, $\theta_0 = 1$, and all terms in $\mathbf{V}$ to zero except V. However, this requires finessing various degeneracies, e.g. wherever $\mathbf{V}$ is inverted. More straightforward is to follow the same process as above, starting from the PIPPET generative model (1) and (2). Either way ultimately yields the PIPPET filter (3).

For multiple event streams j,:

$$dp_t(\mathbf{x}) = \mathcal{L}[p_t(\mathbf{x})]dt + p_t(\mathbf{x})\sum_j(\lambda_j(\mathbf{x})-\mathbb{E}_p[\lambda_j(\mathbf{x})])\cdot(\mathbb{E}_p[\lambda_j(\mathbf{x})]^{-1}dN_j - dt) \tag{25}$$

This follows directly from application of the derivation above to equation (5) in [4] with a discrete spatial dimension. By the methods above, it yields the mPIPPET filter (8) and the mPATIPPET filter:

$$\begin{cases}
d\boldsymbol{\mu}_t = \begin{pmatrix}\bar{\theta}\\ 0\end{pmatrix}dt + \sum_j\left(\hat{\boldsymbol{\mu}}^j-\boldsymbol{\mu}_t\right)\cdot(dN_t^j-\Lambda^j dt) \\
d\mathbf{V} = \begin{pmatrix}2V^{12}+\sigma^2 & V^{22}\\ V^{22} & \sigma_\theta^2\end{pmatrix}dt + \sum_j\left(\hat{\mathbf{V}}^j-\mathbf{V}_t\right)\cdot(dN_t^j-\Lambda^j dt)
\end{cases} \tag{26}$$

References

1. Snyder DL. Filtering and Detection for Doubly Stochastic Poisson Processes. IEEE Transactions on Information Theory. 1972;18(1):91–102. doi:10.1109/TIT.1972.1054756.

2. Harel Y, Meir R, Opper M. A tractable approximation to optimal point process filtering: Application to neural encoding. Advances in Neural Information Processing Systems. 2015;2015-Janua:1603–1611.

3. Eden UT, Brown EN. Continuous-time filters for state estimation from point-process models of neural data. Statistica Sinica. 2008;18:1293–1310.

4. Snyder DL, Fishman P. How to track a swarm of fireflies by observing their flashes. IEEE Transactions on Information Theory. 1975;21(6).
